# Functional characterisation of a novel ovarian cancer cell line, NUOC-1

**DOI:** 10.18632/oncotarget.15821

**Published:** 2017-03-01

**Authors:** Aiste McCormick, Eleanor Earp, Katherine Elliot, Gavin Cuthbert, Rachel O'Donnell, Brian T. Wilson, Ruth Sutton, Charlotte Leeson, Huw D. Thomas, Helen Blair, Sarah Fordham, John Lunec, James Allan, Richard J. Edmondson

**Affiliations:** ^1^ Northern Institute for Cancer Research, Newcastle University, Newcastle upon Tyne, UK; ^2^ Cancer Cytogenetics Department at Newcastle University, Newcastle upon Tyne, UK; ^3^ Northern Gynaecological Oncology Centre, Queen Elizabeth Hospital, Gateshead, UK; ^4^ Institute of Genetic Medicine, Newcastle University, Newcastle upon Tyne, UK; ^5^ Division of Molecular and Clinical Cancer Sciences, Faculty of Biology, Medicine and Health, University of Manchester, St Mary's Hospital, Manchester, UK; ^6^ Department of Obstetrics and Gynaecology, Manchester Academic Health Science Centre, St Mary's Hospital, Central Manchester NHS Foundation Trust, Manchester, UK

**Keywords:** ovarian cancer, clear cell carcinoma, endometrioid carcinoma, mixed histology, cell line model

## Abstract

**Background:**

Cell lines provide a powerful model to study cancer and here we describe a new spontaneously immortalised epithelial ovarian cancer cell line (NUOC-1) derived from the ascites collected at a time of primary debulking surgery for a mixed endometrioid / clear cell / High Grade Serous (HGS) histology.

**Results:**

This spontaneously immortalised cell line was found to maintain morphology and epithelial markers throughout long-term culture. NUOC-1 cells grow as an adherent monolayer with a doubling time of 58 hours. The cells are *TP53* wildtype, positive for PTEN, HER2 and HER3 expression but negative for oestrogen, progesterone and androgen receptor expression. NUOC-1 cells are competent in homologous recombination and non-homologous end joining, but base excision repair defective. Karyotype analysis demonstrated a complex tetraploid karyotype. SNP array analysis of parent and derived subpopulations (NUOC-1-A1 and NUOC-1-A2) cells demonstrated heterogeneous cell populations with numerous copy number alterations and a pro-amplification phenotype. The characteristics of this new cell line lends it to be an excellent model for investigation of a number of the identified targets.

**Materials and Methods:**

The cell line has been characterised for growth, drug sensitivity, expression of common ovarian markers and mutations, clonogenic potential and ability to form xenografts in SCID mice. Copy number changes and clonal evolution were assessed by SNP arrays.

## INTRODUCTION

Epithelial ovarian cancer is often described as the silent killer due to absence of symptoms and late presentation. This combined with the lack of specific sensitive markers and techniques for screening leads to diagnosis at late disease stage in more than 70% of patients. Ovarian cancer is the leading cause of gynaecological cancer mortality worldwide [1] and despite much research into the treatment of ovarian cancer the overall mortality has changed little over the past 20 years with a 5-year overall survival of 30–39% [2]. It has long been recognised by clinicians that ovarian cancer is a set of heterogeneous diseases but, despite this, ovarian carcinoma continues to be treated clinically as a single disease using a combination of debulking surgery and platinum-based chemotherapy. The observed variation in the clinical behaviour of ovarian cancer alongside the growing data reporting molecular heterogeneity suggests that a heterogeneous *in vitro* model for the study of ovarian cancer is long overdue.

Established cell lines provide an invaluable tool for studying biological functions at the molecular and cellular level. Existing human ovarian cancer cell lines possess the advantage of high proliferative capacity, clonogenicity and extended life span in culture. However, ovarian cancer cell lines have rarely been derived from chemotherapy-naive patients, many were established following viral transformation, such as with SV40 Large T antigen or xenograft passaged in immunocompromised mice [3–6]. Very few cell lines are derived from mixed histology tumours and with this type of tumour being less common, reliable models for the study of mixed histology tumours are needed. Additionally, there is evidence to suggest that many cell lines contain significant misidentification, duplication, and loss of integrity [7] making new well characterised models desirable.

In this study, we describe a new ovarian cancer cell line derived from ascites of a chemotherapy-naïve patient. The molecular and growth characteristics of this cell line present unique features, thereby providing the research community with a new tool in the study of different aspects of mixed histology ovarian cancer.

## RESULTS

### Molecular characterization of NUOC-1 cell line

The NUOC-1 cell line was derived from ascites of a chemotherapy naive patient. The female patient was of Caucasian background and 62 years old when she presented with disseminated intraabdominal malignancy. She underwent primary surgery with optimal cytoreduction (with residual milliary disease over the diaphragms and small bowel mesentery). Ascites was collected at the time of surgery for culture. Final histology confirmed FIGO Stage IIIc high grade mixed ovarian carcinoma of the right ovary which was 80% endometrioid, 15% clear cell and 5% high grade serous (HGS) carcinoma. Repeat CT imaging demonstrated new liver and peritoneal metastases and she died of progressive disease 52 days post-op after only 1 cycle of carboplatin. She did not have any known relevant familial history for cancer predisposition.

**Figure 1:**
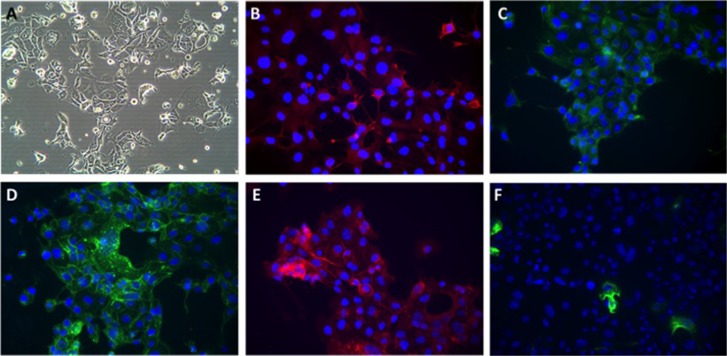
Phenotypic appearances of NUOC-1 cells (**A**) Brightfield demonstrating cobblestone monolayer (20x magnification); immunoflourescent images (40x magnification) with antibodies targeted against: (**B**) Alexafluor 596 anti-CA125; (**C**) FITC-anti-pancytokeratin; (**D**) Alexflour 488 anti-EpCAM; (**E**) Alexafluor 596 anti-MOC 31; (**F**) Alexaflour 596 anti-Vimentin.

**Figure 2:**
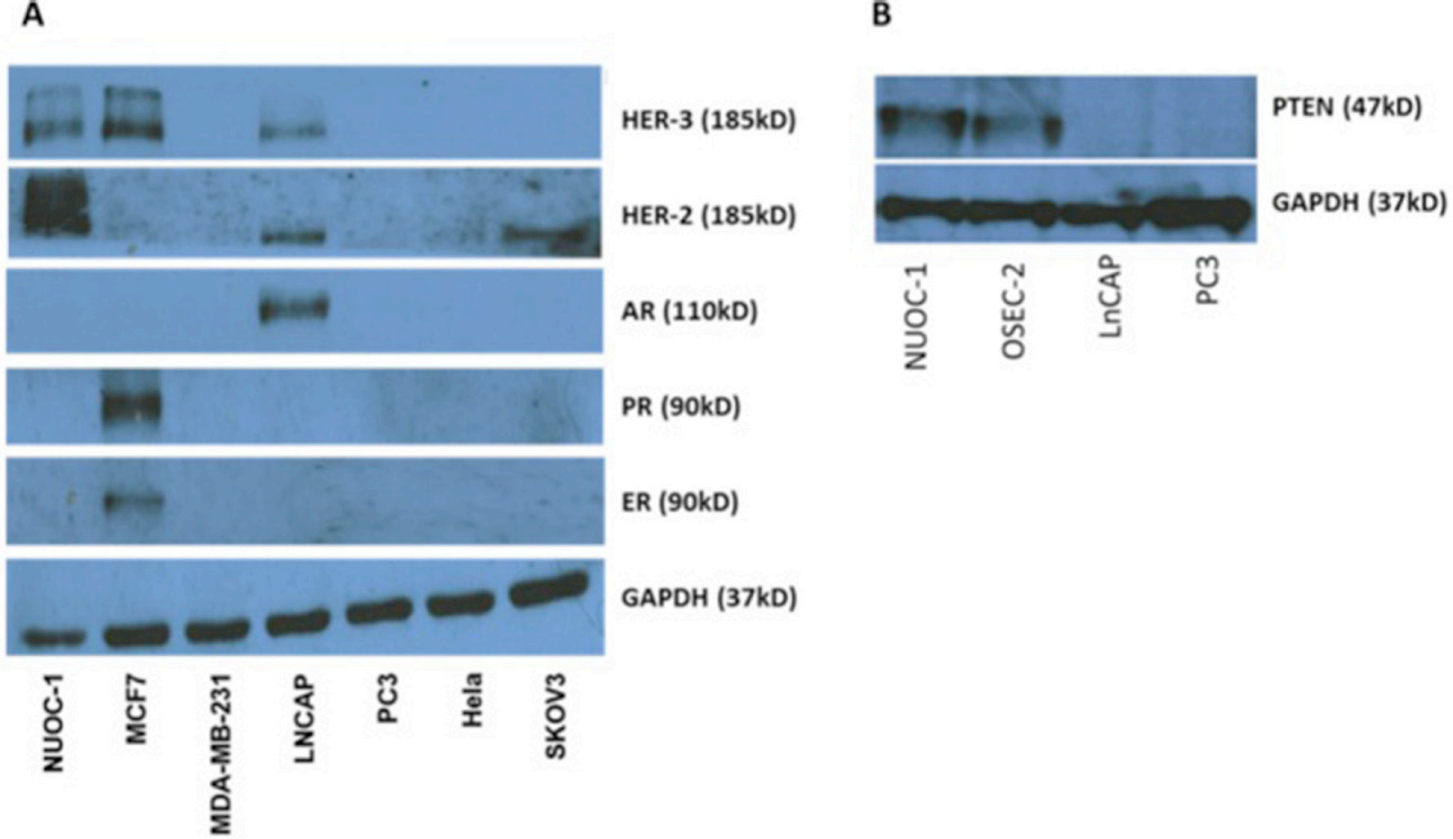
Receptor expression in NUOC-1 cell line assessed by western blotting (**A**) Tyrosine and endocrine receptor expression in the NUOC-1 cell line. NUOC-1 cells express the Her-3 receptor (positive control MCF7) and overexpress the Her-2 receptor (positive control LNCAP and SKOV-3). NUOC-1 cells do not express the oestrogen receptor (positive control MCF7), the progesterone receptor (positive control MCF7) or the androgen receptor (positive control LNCAP). (**B**) PTEN expression levels in NUOC-1 cells demonstrated by Western blot analysis. LNCAP and PC3 cells serve as negative controls whilst OSEC-2 is a positive control. Blots are representative of three independent experiments.

Bright light microscopy revealed a cobblestone morphology characteristic of epithelial cells which was maintained during repeated passage (Figure 1A). The growth of NUOC-1 was initially slow with a 128 hour doubling time, but with continued culture this stabilised to 58 hours. NUOC-1 formed colonies when grown on plastic at an efficiency of 2.2% +/− 0.6%. NUOC-1 cells stained positive for proteins characteristic of epithelial ovarian carcinoma (pancytokeritin, epithelial cell adhesion molecule (EpCAM), epithelial related antigen MOC31 and cancer antigen 125 (CA125)) and negative for germ cell related antigen D240 and Vimentin (Figure 1B–1F). NUOC-1 cells did not express oestrogen, progesterone and androgen receptors (Figure 2A), but expressed the receptor tyrosine-protein kinase erbB-3 (HER-3) receptor and the receptor tyrosine-protein kinase erbB-2 (HER-2) receptor at a higher level than LNCAP and SKOV-3 cell line controls (Figure 2A). Sequencing of the entire coding region of *BRCA1*, *BRCA2* and *phosphatase and tensin homologue deleted on chromosome 10* (*PTEN*) revealed no germline or somatic mutations. Furthermore PTEN protein expression was confirmed by western blotting (Figure 2B).

### P53 function assessment

**Figure 3:**
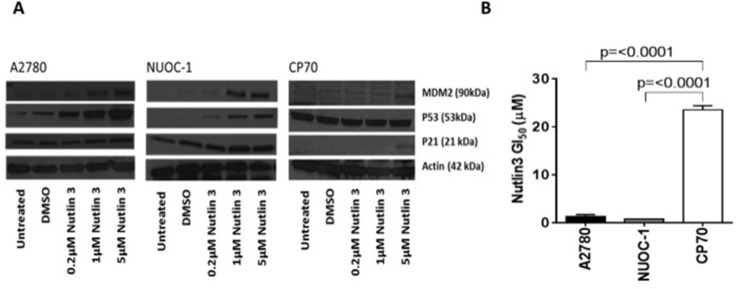
p53 function in NUOC1 cells (**A**) Activation of MDM2, p53 and p21 protein in response to Nutlin3 treatment assessed by western blotting. Images are representative of 3 independent experiments. (**B**) Growth inhibition induced by Nutlin3 treatment in NUOC-1 cell line assessed by SRB assay. The inhibition in NUOC-1 is compared to p53 wild type A2780 and p53 mutant CP70 cell lines. Results are the average of 3 independent experiments repeats. Error bars are SEM.

Dysregulated tumour protein p53 is recognised as a characteristic feature of HGS. To determine if the small proportion of the HGS within NUOC-1 immortalised, p53 status was determined. Sanger dideoxy sequencing of *TP53* Exons 3-9 detected no mutations. Consistently with functional p53, treatment with Mouse double minute 2 homolog (MDM2)-p53 antagonist (Nutlin-3) demonstrated accumulation of MDM2 in NUOC-1 cells concomitant with a dose dependent increase in p53 and cyclin-dependent kinase inhibitor 1 (p21) protein levels (Figure 3A). In contrast, Nutlin-3 treatment of CP70 cells did not significantly induce p53 or p21, consistent with the established *TP53* mutation in this cell line. Nutlin-3 had a greater growth inhibitory effect in *TP53* wild-type NUOC-1 cells compared to *TP53* mutant CP70 cells (GI_50_ 0.7 μM +/−0.03 μM vs 23.5 μM +/−0.9 μM; *p* < 0.00001, Figure 3B).

### DNA repair assessment

**Figure 4:**
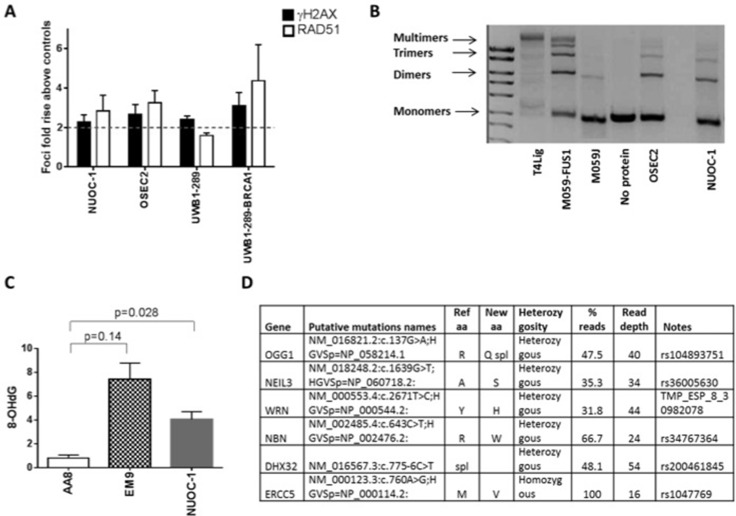
DNA repair capacity of NUOC-1 cell line (**A**) Homologous recombination function assessed by RAD51 foci formation after 24 hour treatment with 10 μM Rucaparib compared to DMSO controls assessed by immunofluorescence. Fold rise in RAD51 foci in control and treated samples. Results are the average of foci counted across 3 separate microscopic fields of view counting > 50 cells in each. Error bars are SD. (**B**) Non homologous end joining function assessed by NUOC-1 cell extracts ability to rejoin linearised vectors into multimers. Images are agarose gels stained with GelRed. DNA-PK mutated M059J cells were used as negative control and DNA-PK competent M059J-FUS-1 cells were used as positive controls for end joining. Images are representative of 3 experimental repeats. (**C**) Base excision repair of NUOC-1 assessed by competitive ELISA. Results are the measurement of 8-OHdG levels. AA8 (BER proficient) cell line was used as positive control, EM9 (BER deficient) cell line was used as a negative control. (**D**) Summary of genetic data. The putative pathogenic variants (known mutations or variants with a population frequency of < 1 : 1000) are shown.

DNA repair ability of NUOC-1 cells was assessed by validated functional assays and sequencing of DNA repair genes. A greater than two fold increase in γH2AX and RAD51 foci formation following exposure to IR is used as a cut off to define homologous recombination (HR) competence and a < 2 fold increase in RAD51 is classed as HR deficient [8, 9]. NUOC-1 cells were deemed HR competent with a 2.84 fold rise in RAD51 foci, compared to untreated controls (Figure 4A). Non homologous end joining (NHEJ) function is assessed by the ability of cell extracts to rejoin incompatible vector DNA ends correctly. NUOC-1 cells were found to be NHEJ competent as evidenced by the ability to rejoin both compatible and incompatible DNA breaks correctly (Figure 4B). Excess production of 8-OHdG inferred the non-functioning of base excision repair (BER) and was quantified by competitive ELISA in NUOC-1 cells (Figure 4C). AA8 (BER competent) with its derivative EM9 cell lines (BER deficient due to XRCC1 mutation) were used as positive and negative controls. NUOC-1 cell 8-OHdG level was 4.0 nM +/− 0.64 nM, indicating that NUOC-1 cells were BER defective (Figure 4C). Massively parallel sequencing identified heterozygous mutations in *OGG1*, *WRN*, *NBN* and *NEIL3*, and homozygous mutation were identified in *ERCC5* (Figure 4D).

### Drug sensitivity assessment

Sensitivity to common therapeutics was assessed by SRB growth inhibition assays and compared to BRCA1 mutant HGSC cell line, UWB1-289 and for assessment of toxicity to normal tissue, a cell line derived from normal ovarian surface epithelium (OSEC-2). NUOC-1 cell line was significantly more resistant to rucaparib compared to UWB1.289 cells (*p* < 0.0001, Table 1), with GI_50_ similar to OSEC-2 cells. NUOC-1 cells were also significantly more resistant to cisplatin when compared to UWB1.289 (*p* < 0.0001, Table 1), but more sensitive than OSEC-2 cells (*p* = 0.001, Table 1). Compared to the OSEC2 cell line, NUOC-1 was more sensitive to camptothecin (*p* < 0.0001, Table 1), no significant difference was noted when compared to UWB1.289 cells. NUOC-1 cells were more resistant to Paclitaxel compared to OSEC-2 cells (*p* = 0.017, Table 1 ) and UWB1.289 cells (*p* = 0.007, Table 1). In contrast, there was no significant difference between NUOC-1, UWB1.289 and OSEC2 in terms of sensitivity to doxorubicinor irradiation (Table 1).

**Table 1:** Sensitivity to cytotoxic agents in NUOC-1 cells compared to UWB1.289 and OSEC2 cell line

Cytotoxic agent	Mean GI50 and 95% confidence interval				
	NUOC-1	UWB1-289	*F* test *p* =	OSEC-2	*F*-test *p* =
Cisplatin (μM)	1.240.93 to 1.66	0.120.07 to 0.22	< 0.0001	2.461.78 to 3.39	0.001
Paclitaxel (nM)	217.1154.3 to 305.4	54.1747.75 to 61.45	0.007	97.9372.97 to 131.0	0.017
Camptothecin (nM)	15.859.10 to 27.63	6.983.04 to 16.03	0.59	171.0103.0 to 283.8	< 0.0001
Doxorubicin (nM)	38.8331.77 to 47.46	27.3716.28 to 46.03	0.66	37.6828.73 to 49.41	0.87
Rucaparib (μM)	6.184.69 to 8.15	0.620.31 to 1.23	< 0.0001	5.313.91 to 7.21	0.16
Irradiation (Gys)	3.552.43 to 5.18	2.691.65 to 4.37	0.94	4.943.25 to 7.49	0.21

Results are mean GI_50_ and 95% CI in μM or nM assessed by SRB assay. Results are the average of 3 independent experiments with 6 experimental repeats in each. Results were compared using comparison of fits for non linear regression, with Bonferroni correction for multiple comparisons.

### Clonal evolution and copy number alterations

**Figure 5:**
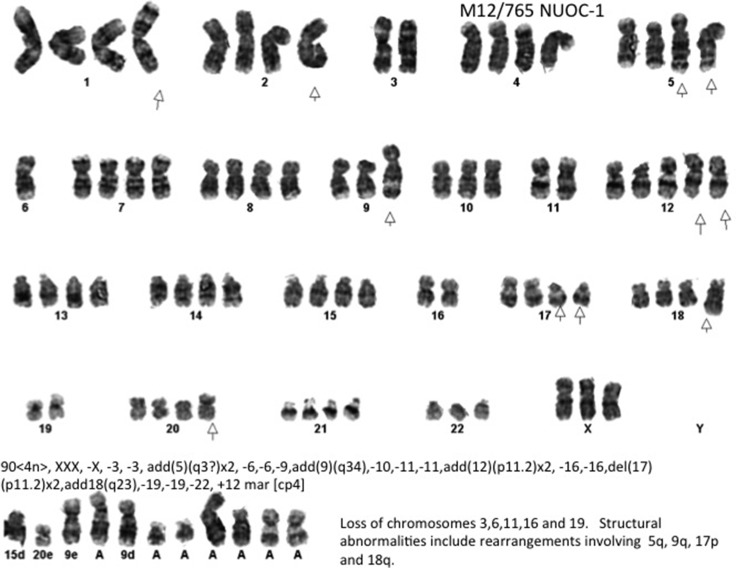
Representative G-banded metaphase Arrows indicate the aberrations noted in all metaphases assessed. The result is composite of 4 metaphases assessed.

NUOC-1 cells revealed a complex, near-tetraploid karyotype, with loss of chromosomes 3,6,11,16 and 19, and structural abnormalities including rearrangements of 5q, 9q, 17p and 18q (Figure 5).

To assess clonal evolution two NUOC-1 subpopulations were derived. NUOC-1 cells were split at passage 4 and either grown continuously to passage 14 (NUOC-1-A1) or frozen and stored in liquid nitrogen for 12 months before being thawed and also grown to passage 14. Short tandem repeat (STR) profiling of the parental NUOC-1 cells and the two sub-clones show that all three cell lines have identical profiles at 8 independent STRs (Supplementary Table 1).

**Figure 6:**
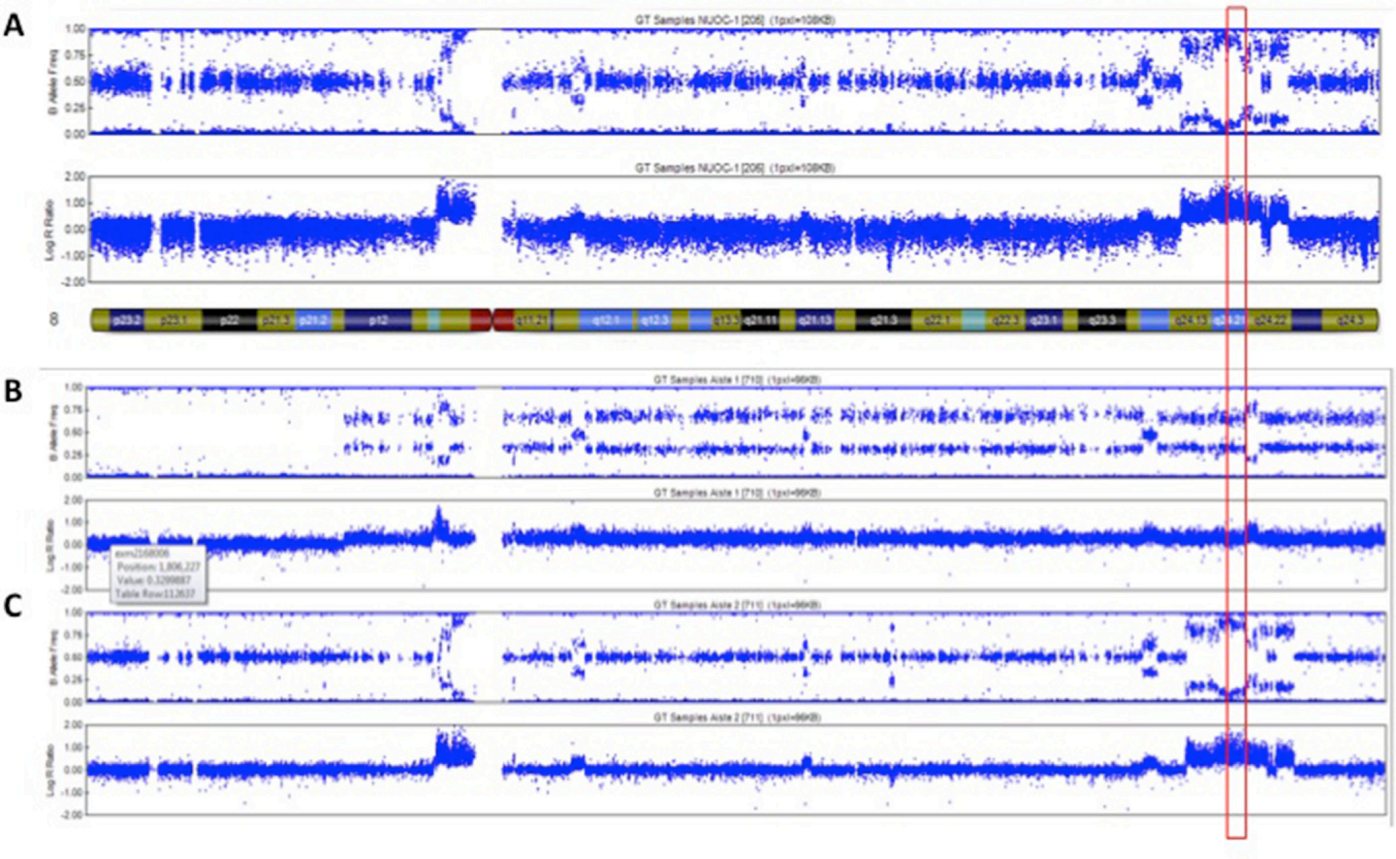
Copy number profile of chromosome 8 in (**A**) NUOC-1, (**B**) NUOC-1-A1 and (**C**) NUOC-1-A2 cell lines. Each SNP marker is represented and aligned to its position on the chromosomes as well as its designated copy number state. An ideogram of chromosome 8 is positioned below the SNP marker plots. Red square – location of *c-MYC* gene.

**Figure 7:**
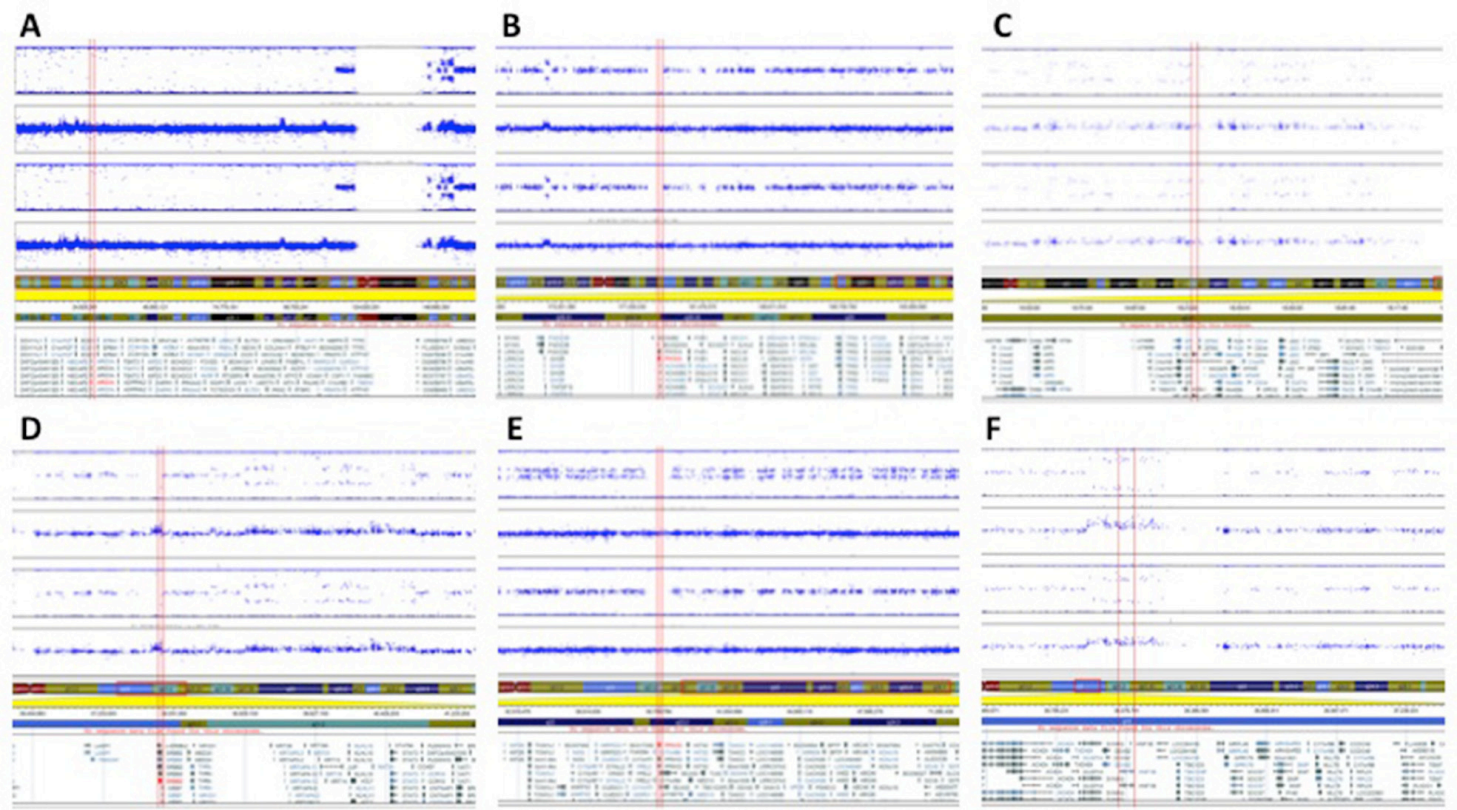
Copy number profiles of NUOC-1-A1 and NUOC-1-A2 cell lines showing Log R ratio and B allele frequency Each SNP marker is represented and aligned to its position on the chromosomes as well as its designated copy number state. Ideograms of each chromosome are positioned below the SNP marker plots. (**A**) *ARID1A* captured by a large region of copy neutral LOH on chromosome 1. (**B**) *PIK3CA* located in a region of copy neutral LOH. (**C**) *AKT1*, captured by a 155Kb amplicon on chr 14. (**D**) *ERBB2* (HER2), captured by amplicon of 405Kb on chr 17. (**E**) *PPM1D* located in a region of apparent copy neutral LOH. (**F**) *HINF1B*, captured by amplicon of 115Kb on chr 17.

Intra-chromosomal copy number alterations deviating from the baseline copy number state (tetrapoloid) were identified in NUOC-1-A1, NUOC-1-A2 and parental NUOC-1 cells using OmniExpressExomeBeadChip genotyping data. NUOC-1-A1 and NUOC-1-A2 carried numerous common copy number alterations, consistent with a shared recent ancestry (Supplementary Table 2). However, each cell line also carried a small number of unique copy number alterations not seen in the other cell line, indicating ongoing genomic evolution. Greater than 95% of copy number alterations were gains, with an average amplicon size of 2.6 Mb and 1.2 Mb in NUOC-1-A1 and NUOC-1-A2, respectively. The vast majority of copy number alterations shared by NUOC-1-A1 and NUOC-1-A2 were also visible in parental NUOC-1 cells. With regard to the unique copy number alterations, NUOC-1-A1 has a low level copy number gain affecting the long arm and some of the short arm of chromosome 8 that is not seen in NUOC-1-A2. However, NUOC-1-A2 has a complex high level amplification on chromosome 8 that captures the *MYC* locus (Figure 6), and which is present in the dominant clone. Other genes implicated in type I ovarian cancer pathogenesis are also affected by copy number alterations in NUOC-1-A1 and/or NUOC-1-A2 (Figure 7A–7F). For example, the *HINF1B* and *ERBB2* genes are captured by amplicons of 405Kb and 115Kb, respectively, on chromosome 17 in both NUOC-1-A1 and NUOC-1-A2. Likewise, the *AKT1* gene is captured by a 155Kb amplicon on chromosome 14. The *ARID1A* gene is captured by a large region of copy neutral loss of heterozygosity on chromosome 1.

### Assessment of *MYC* amplification

**Figure 8:**
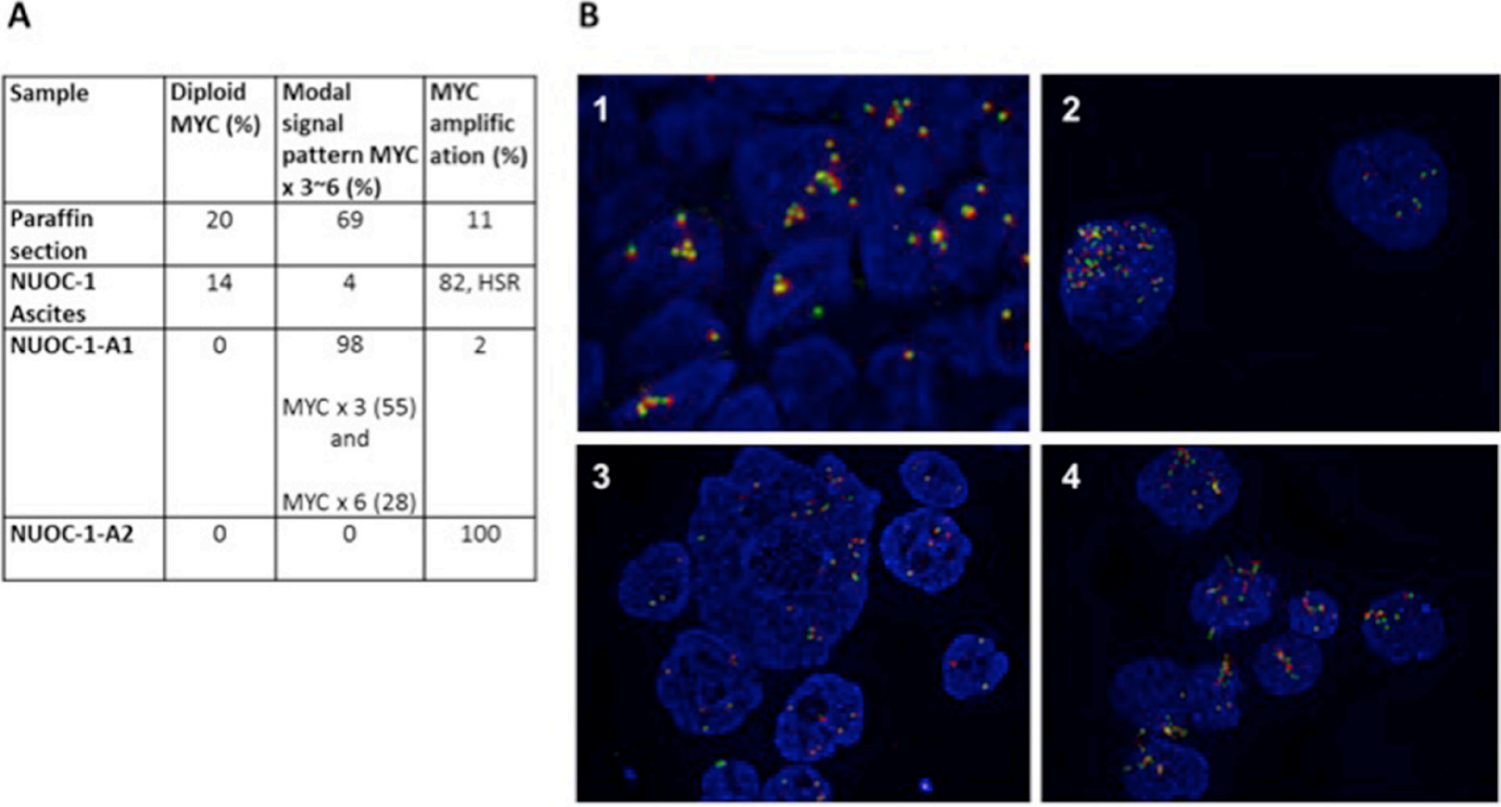
FISH for MYC results for NUOC-1 MYC was assessed in paraffin embedded tumour sample from the patient NUOC-1 cell line was derived, ascites sample, *NUOC-1-A1 and NUOC-1-A2 cells. (***A**) *The percentage of normal, increased chromosome and MYC amplified cells is stated. HSR - homogeneously staining regions*. (**B**) FISH immunoflourescent images. The Cytocell MYC ‘breakapart’ Probe set was hybridised to nuclei as recommended by the suppliers. Images were taken at 40x magnification. Results are 1. FFPE embedded tumour sample, 2. NUOC-1 ascites sample, 3. NUOC-1-A1, 4. NUOC-1-A2.

Fluorescence *in situ* hybridization (FISH) for *MYC* copy number was carried out in samples at four stages of cell line development (Figure 8). FFPE sections of tumour identified a modal *MYC* signal pattern showing copy number gain (3-6 copies) in 69%, *c-MYC* amplification in 11% and diploid *MYC* signal in 20% of interphase cells analysed. Ascites sample contained 14% diploid MYC expressing cells. The majority of cells contain *MYC* amplification (82%) with a small number showing copy number gains (4%). This finding demonstrates heterogeneity between ascites and solid tumour. The differences of *MYC* between NUOC-1-A1 and NUOC-1-A2 closely relate to the findings from the SNP array. Mostly copy number gains were detected in NUOC-1-A1 (98%), which would be seen as normal copy number in the SNP array, in comparison to 100% *MYC* amplification observed in NUOC-1-A2. Also, the results support the hypothesis that NUOC-1-A2 forms the major clone, and NUOC-1-A1 the minor clone, in the parental cell line. No separation of *MYC* probes was seen in cells with increased chromosome numbers, indicating that *MYC* translocation was not present. In cells with amplification, the signal patterns suggested the presence of one or more homogeneously staining regions. In some cells isolated amplification of the 5′ components of the MYC probe set were seen suggesting the presence of varied size amplicons.

### *In vivo* tumourigenicity

Finally, we assessed the potential of *in vivo* growth by injecting tumour cells at intraperitoneal or subcutaneous sites in nude mice. No subcutaneous or intraperitoneal tumours or ascites formation was observed after 100 days (data not shown).

## DISCUSSION

Here we describe the establishment and characterization of a novel ovarian cancer cell line derived from a chemotherapy naïve patient. Extensive characterisation to date and the unique features described in this cell line make it a novel tool for ovarian cancer research.

In order to establish new cellular models of ovarian cancer, all samples of ovarian tissue were processed to derive primary cell cultures as previously described [10]. Primary ovarian cultures provide a model which better represents the tremendous heterogeneity of ovarian cancer, but which have a number of limitations. Specifically, primary cultures have a very short life span and slow growth, which limits characterisation as well as their application in repeat experiments. Of the 156 primary cultures we have established so far, NUOC-1 is the only culture to spontaneously immortalise. NUOC-1 cells continue to maintain their morphology and epithelial marker expression over repeated passages.

NUOC-1 was derived from ascites of a mixed histology tumour. *TP53* mutations are reported in the majority of HGS ovarian cancer [11], but are rare in endometrioid / clear cells cancers. Therefore the *TP53* wildtype genotype of NUOC-1 is consistent with type I ovarian cancers. Furthermore, other genes implicated in type I ovarian cancer pathogenesis, namely PIK3CA, HINF1B, ERBB2 (HER2), AKT1, PPM1D, and ARID1A, are also affected by copy number alterations in NUOC-1. Therefore, the NUOC-1 cell line is also representative of endometrioid / clear cell ovarian carcinoma. Furthermore, NUOC-1 represents a useful model for investigations of PTEN function, DNA repair as well as hormone receptor negative and tyrosine receptor positive phenotypes.

The results obtained from G-banding karyotype are consistent with previously published karyotype studies on epithelial ovarian cancer where high genomic instability is observed [12]. The heterogeneity of cells observed in NUOC-1 better reflects the heterogeneity of ovarian cancer that many cell lines which were derived from a single clonal population lack. SNP array results provide further insight into the extensive genomic alterations present in this cell line. Mixed histology tumours are common in ovarian cancer. There remains a debate as to whether these are monoclonal tumours with heterogeneous morphology or truly mixed tumours. Most recent data would tend to lean towards a monoclonal origin [13].

There is a significant difference in the genomic pattern of the two NUOC-1 sub-cultures, and this is different from the parental line. NUOC-1 cells are susceptible to changes in copy number across the entire genome, and most of these changes were gains rather than losses. This instability could give rise to a very heterogeneous tumour in many features, including basic cell morphology and is perhaps more likely to develop into a mixed morphology tumour. An alternative hypothesis is that the tumour contains different pre-existing clones which have grown into the two subcultures. Although the two sub-lines differ from each other and also from the parent population they share most of their features. Despite several months in culture the two sub-clones retain a genetic profile, in terms of both somatic copy number alterations and microsatellite repeat length that is very similar to the parental cell population from which they were derived. The parental population was found to be heterogeneous and continuous selection in different flasks has led to the outgrowth of two different sub-clones, with the NUOC-1-A1 sub-clone being derived from the major clone in the parent population. Although the novel cell lines described here are clearly susceptible to the acquisition of somatic copy number alterations, the evidence suggests that the cells are sufficiently stable in long-term culture to justify their use as an experimental model.

NUOC-1 cells were not able to form xenografts in mice which was consistent with the poor clonogenicity on plastic and in soft agar. It has been previously suggested that cell lines derived from patients with indolent disease exhibit low tumourgenicity. This is not the case for the NUOC-1 cell line, as the patient from which the cell line originated had extremely aggressive disease and lived only 52 days post optimal debulking surgery. Whilst the inability of NUOC-1 cells to form xenografts limits its use in xenograft models, this should not detract from the unique phenotype of this cell line.

## MATERIALS AND METHODS

### Sample and patient data

Ethical approval was granted (12/NW/0202) for the collection of ascites from consented patients undergoing surgery for ovarian cancer at the Queen Elizabeth Hospital, Gateshead, UK. Clinical details were recorded and specimens registered and handled in accordance with the Human Tissue Act. Samples were assigned a reference number to retain anonymity. Histopathology and tumour grades were assigned by a pathologist according to the International Federation of Gyneacology and Obstetrics (FIGO) criteria. Primary cultures were generated and maintained as previously described [10]. Briefly 20 ml of ascites was added to 20 ml of warmed Sigma RPMI 1460 HEPES modified culture medium supplemented with 20% v/v foetal calf serum and 100 μl/ml penicillin and streptomycin in T75 flasks and incubated at 37°C, in 5% CO_2_ humidified air. Cells were passaged, frozen and thawed as previously described [14].

### Cell lines

All cell lines unless stated otherwise were grown in RPMI 1640 media supplemented with 10% FBS and 100 units/ml penicillin/streptomycin incubated at 37°C in 5% CO_2_. A2780, a human ovarian carcinoma cell line and CP70, a mismatch repair (MMR) deficient and *TP53* mutant variant of A2780, 5-fold resistant to cisplatin relative to the parental A2780, were a kind gift from Prof. R. Brown (formerly of the Cancer Research UK Beatson Laboratories, Glasgow). The SKOV-3 cell line derived from a human ovarian adenocarcinoma, PC3 and LNCAP prostate cancer cell lines, MCF7 and MDA-MB-231 breast cancer cell lines and Hela is a cervical cancer cell line were all purchased from the American Type Tissue Culture Collection (ATCC, VA, USA).

M059J, DNA-PKcs-deficient human glioblastoma cells, were grown in DMEM supplemented with 10% FBS, 100 units/ml penicillin/streptomycin. M059FUS1 (M059J transfected with a portion of chromosome 8 carrying the DNA-PKcs gene) cells were cultured in full media with 400 μg/ml G418. Both were purchased from ATCC.

OSEC2 cells developed at Newcastle University from normal ovarian surface epithelium were incubated at 33°C.

UWB1-289 is a BRCA1-null human epithelial ovarian cancer cell line derived from a high grade serous ovarian carcinoma and was cultured in 50% RPMI 1640 media supplemented with 10% FBS, 100 units/ml penicillin/streptomycin and 50% (v/v) MEBM BulletKit media (Lonza) supplemented with 10% FBS. UWB1-289-BRCA1 is derived from UWB1-289 cells in which BRCA1 was restored and was cultured in full media with 400 μg/ml G418; both were obtained from ATCC.

### Characterisation

Morphological features were studied under an Olympus CK40 inverted microscope at 40 x magnification. Images were captured using VisiCam® software (VWR, USA).

### Immunofluorescence

Standard techniques for immunofluorescence were used to stain for pancytokeritin (mouse monoclonal anti-pancytokeratin FITC–conjugated antibody, Upstate Millipore Corp., USA), EpCAM (mouse monoclonal anti-CD326 Alexafluor® 488–conjugated antibody, Biolegend, USA), CA125 (mouse monoclonal anti-CA125 antibody, Abcam, USA, and Alexafluor^®^ 546 goat anti-mouse secondary antibody, Invitrogen, USA), MOC-31 (mouse monoclonal anti-MOC-31 antibody, Dako, Germany, and Alexaflour^®^ 596 goat anti-mouse secondary antibody, Invitrogen, USA), D2-40 (mouse monoclonal anti-D2-40 antibody, Dako, Germany, and goat anti-mouse Alexafluor^®^ 596 secondary antibody, Invitrogen, USA) and vimentin (rabbit monoclonal anti-vimentin antibody, clone EPR3776, Abcam, USA, and Alexafluor^®^ 488 goat anti-rabbit secondary antibody, Invitrogen, USA).

### Homologous recombination assay

Cells were seeded onto glass cover slips and treated with 2 Gy ionising radiation and rucaparib at 10 μM for 24 hours to induce DNA damage. All experiments were performed alongside untreated controls with equivalent 0.1% DMSO. Cells were then fixed and rehydrated prior to staining with 1:100 mouse monoclonal anti-γH2AX (Upstate, Millipore Corp., USA) and 1:100 goat polyclonal anti-Rad51 (Calbiochem, EMD Biosciences, Inc., USA) antibodies with appropriate secondary fluorochrome conjugated antibodies, as previously described [8]. Image J counting software [15, 16] was used to count γH2AX and RAD51 nucleic foci. Cells were classed as homologous recombination (HR) competent if there was more than a 2 fold increase in RAD51 foci, after DNA damage was confirmed by a 2 fold increase in γH2AX.

### Non homologous end joining assay

Cell extracts were prepared as previously described [17] using three T175 flasks for each lysate. Trypsinised cells were homogenized in hypotonic buffer (final volume 0.5 ml) and mixed with 0.5 vol of high salt buffer. Ultracentrifugation (Beckman Optima TL120) was performed for 56 min at 70000RPM at 4°C using thick-walled polyallomer microfuge tubes in a Beckman TLA120.2 rotor to provide a protein extract. Vectors, which on digestion with BstXI yielded a 3.2 kb plasmid and 1.2 kb λ fragment with either compatible or 2 bp incompatible ends provided substrates and controls for the NHEJ assay, were kindly donated by Dr Anne Kiltie (Oxford, UK). DNA fragments were gel-purified using spin columns (Qiagen, UK) and resuspended to 25 ng/μl. End-joining reactions (20 μl) were carried out with 45 μg protein extract and 50 ng DNA substrate in the presence of 50 mM HEPES pH 8.0; 40 mM KOAc; 0.5 mMMg(OAc)_2_; 1 mM ATP; 1 mM DTT and 0.1 mg/ml BSA at 37°C for 2.5 hr. Samples were incubated with RNaseA (80 μg/ml) for 10 min and then protein was removed by incubation with proteinase K (2 mg/ml) and 0.5% (w/v) SDS for 10 min at 37°C followed by 10 min 55°C and 10 min 65°C incubation. Plasmid DNA was extracted with Tris-buffered phenol/chloroform/isoamyl alcohol. Analysis of plasmid end joining was performed by agarose (0.7%) gel electrophoresis and GelRed (VWR) staining. Images were collected and quantified using G-BOX.

### Sulforhodamine B (SRB) assay

A routine sulforhodamine B (SRB) assay was used to assess cytotoxicity and cell growth as previously described [18]. Briefly, cells were seeded at a concentration of 1000 cells/well of a 96-well plate and after adherence, treated with various concentrations of inhibitors for 10 days before fixation, staining and spectrophotometer assessment. Inhibitors used were cisplatin, paclitaxel, camptothecin, doxorubicin, rucaparib (donation from Clovis), Nutlin3 and irradiation (in house D3300 X-ray system). For assessment of growth, cells were fixed daily for 14 days and the doubling time was calculated according to the slope of the linear portion of the growth curve.

### Colony formation assays

50,000 cells/well were seeded in a 6 well plate for 24 hours. Cells were then incubated for 24 hours with various concentrations of the cytotoxic agent before reseeding at concentrations of 2,500, 5,000 and 10,000 cells for each drug treatment. Cells were incubated at 37°C for 30 days. The medium was aspirated, plates were washed in PBS and then fixed using Carnoy's fixative (acetic acid: methanol 1:3 v/v) followed by staining with 1% crystal violet.

### *In vivo* growth in SCID mice

All animal studies were performed in compliance with the UK Home Office Animals (Scientific Procedures) Act 1986 for the use of animals in scientific procedures and have undergone local ethical review. Project licence number PPL 60/42222. The tumorigenic potential of cell line was assessed based on their ability to form tumours in 8–10 week old female nude SCID mice at subcutaneous (s.c.) right gluteal or intraperitoneal (i.p.) injection sites (cells were first labelled by transfection with a luciferase containing vector, which was kindly gifted by Dr A Elder [Newcastle upon Tyne, UK]). Five mice were transplanted with 5 × 10^6^ cells suspended in 50% medium / 50% v/v matrigel subcutaneously and five mice were transplanted with luciferase-expressing 5 × 10^6^ cells suspended in phosphate buffered saline (PBS) for i.p. injections. The animals were housed under sterile conditions in a laminar flow environment with unrestricted access to food and water. Tumour formation was assessed by observation of subcutaneously implanted mice for 100 days. The five i.p transplanted mice underwent non-invasive whole-body imaging starting at 0, 10 and 30 days after implantation using the IVIS Spectrum Imaging system (Caliper Life Sciences, Hopkington, MA, USA). Mice were injected i.p. with 3 mg/mouse D- luciferin (Promega) solution ten minutes before being anaesthetized for the imaging procedure. Photon emission was captured and expressed in p/s/cm2/sr using Living Image software (Version 4.3.1., Caliper Life Sciences).

### Western blotting

Cells were lysed using Merck phosphosafe buffer (Calbiochem) and protein concentration was quantified using a Pierce protein assay (ThermoScientific) as per manufacturer's instructions. Samples were denatured at 100°C for 10 minutes before separation of protein in the cytosolic extracts according to size using SDS-PAGE gel electrophoresis. Proteins were transferred electrophoretically from the gels onto a nitrocellulose membrane (Hybond C Membrane (GE Healthcare). Antibodies used for detection of protein were: Oestogen receptor a 1:1000 (Santa Cruz 8005), Progesterone receptor 1:1000 (Cell signalling), Androgen receptor 1:1000 (Santa Cruz 7305), HER-2 1:1000 (Santa Cruz 33684), HER-3 1:1000 (Santa Cruz 285), MDM2 3:1000 (Calbiochem), p53 2:1000 (Vector), p21 1:100 (Calbiochem), GAPDH 1:3000 (Santa Cruz) and Actin 1:1000 (Sigma).

### Mutation analyses

*TP53* mutations were detected by Sanger dideoxy DNA sequencing. Polymerase chain reaction (PCR) was used to amplify exons 3–9 of the *TP53* gene in GeneAmp PCR System 9700 (Applied Biosystems). PCR was performed in a 25 μl volume containing 200 ng of DNA; 1 × PCR Gold buffer (New England BioLabs); 2.5 nmol each dNTP, 1.5 mM MgCl2; 15 pmol of each primer (Sigma-Aldrich); and 1.25 U of Amplitaq Gold (New England BioLabs). The PCR conditions were 5 min at 95°C, 40 cycles (30 s 94°C, 30 s 55–60°C, 30 s 72°C). PCR products were then purified according to the manufacturer's instructions using a PureLink PCR Purification Kit (Invitrogen, Cat no: K3100-01/-02). Mutations were confirmed by sequence analysis by DBS Genomics (Durham University, UK). Sequencing results were analysed using the SeqMan software package (DNA star).

### Next-generation sequencing

Genomic DNA extracted from cancer cells cultured from NUOC-1 was sheared to a mean length of 500 bp using nitrogen nebulisation. A custom-made NimblegenSeqCap EZ library (Nimblegen, Madison, WI, USA) was used to enrich for the sequence of interest. This comprised the exonic regions of > 180 DNA repair genes and limited intronic material. Captured sequence was subjected to massively parallel next-generation DNA sequencing by using the Roche 454 GS FLX platform (454 Life Science, Branford, CT, USA). A bespoke analysis pipeline was used to identify pathogenic changes and potential variants of interest (single nucleotide polymorphisms with < 1% population prevalence) across the target genes.

### STR profiling

STR profiling was perfomed by NewGene Limited. Briefly eight short tandem repeat (STR) loci, plus Amelogenin, were amplified using the GenePrint^®^ 10 System, supplied by Promega. The reaction products were processed using an Applied Biosystems^®^ 3130 × l Genetic Analyzer and the resulting data interpreted using GeneMarker^®^ v2.6.0 software (SoftGene LLC). Appropriate positive and negative controls were included in every batch of samples analysed.

### SNP array copy number analysis

For SNP array analysis DNA was extracted using a QIAmp DNA Kit (Qiagen) as per the manufacturer's instructions. Intra-chromosomal copy number alterations were determined using data derived from NUOC-1-A1 and NUOC-1-A2 cell lines genotyped using the OmniExpressExome BeadChip from Illumina (San Diego, California). Copy number variation data was analysed using cnvPartition v.3.2.0 in GenomeStudio V2011.1 (Illumina), with deviations from copy neutral called if affecting 10 or more markers and achieving a confidence threshold score of at least 50. Alterations affecting the X chromosome and all 22 autosomal chromosomes were characterised. Deviations from copy neutral called by cnvPartition were confirmed manually (It should be noted that copy neutral equates to a copy number of 4 for NOUC-1, NUOC-1-A1 and NUOC-1-A2 because these cell lines are predominantly tetraploid, as determined by G-banding karyotyping (Figure 5)). A small number of additional copy number alterations not identified by cnvPartition were identified manually. Regions of copy neutral loss of heterozygosity (LOH) exceeding 10 Mb were also identified manually.

Parental NUOC-1 cells were also genotyped using the OmniExpressExome BeadChip and the resulting data visualised using GenomeStudio V2011.1. These data were manually interrogated to confirm whether copy number alterations and regions of copy neutral LOH identified in NUO-C1-A1 and NUOC-1-A2 were also visible in parental NUOC-1 cells. Regions of copy number alteration and copy neutral LOH identified in NUOC-1-A1, NUOC-1-A2 and parental NUOC-1 cells are detailed in Supplementary Table 2.

### G-band karyotyping

Karyotyping was performed by the Cancer Cytogenetics department at Newcastle Hospitals NHS Foundation Trust, according to established protocols. Metaphase chromosome spreads were prepared by incubating proliferating cells with 100 ng/ml colcemid for 4 h followed by resuspension in 75 mM KCl for 7 min. Cells were fixed by resuspension in 3:1 methanol:acetic acid before karyotyping. Slide-fixed cells were incubated overnight at 60°C and G-banded by standard trypsin and Giemsa methods. Four metaphase chromosome spreads were analysed for each population and the karyotypes recorded.

### Fluorescence *in Situ* Hybridization for c-MYC

FISH analysis was performed by the Cancer Cytogenetics department at Newcastle Hospitals NHS Foundation Trust, according to established protocols. The Cytocell MYC ‘breakapart’ Probe set was hybridised to nuclei as recommended by the suppliers. Slides were heated to 72°C for 5 min and then incubated for 24 hr at 37°C in a humidified hybridisation chamber (HYBrite; Abbott Molecular). After hybridisation, slides were counterstained with 4′,6-diamidino-2-phenylindole (Vector Laboratories, Peterborough, UK). FISH was scored with an Olympus BX-61 fluorescence microscope (Olympus, Southend-on-Sea, UK) with a × 100 oil objective. Images were analysed using the CytoVision 7.2 SPOT counting system (Leica Microsystems, Gateshead, UK). A minimum of 100 nuclei were scored per test by two independent analysts.

## SUPPLEMENTARY MATERIALS FIGURES AND TABLES





## References

[1] Statistics NOf (2011). Mortality statistics: Deaths registered in 2010 (Series DR) Table 5.2.

[2] Parmar MK, Ledermann JA, Colombo N, du Bois A, Delaloye JF, Kristensen GB, Wheeler S, Swart AM, Qian W, Torri V, Floriani I, Jayson G, Lamont A (2003). Paclitaxel plus platinum-based chemotherapy versus conventional platinum-based chemotherapy in women with relapsed ovarian cancer: the ICON4/AGO-OVAR-2.2 trial. Lancet.

[3] Auersperg N, Maines-Bandiera SL, Dyck HG, Kruk PA (1994). Characterization of cultured human ovarian surface epithelial cells: phenotypic plasticity and premalignant changes. Lab Invest.

[4] Nitta M, Katabuchi H, Ohtake H, Tashiro H, Yamaizumi M, Okamura H (2001). Characterization and tumorigenicity of human ovarian surface epithelial cells immortalized by SV40 large T antigen. Gyn Onc.

[5] Shenhua X, Lijuan Q, Hanzhou N, Xinghao N, Chihong Z, Gu Z, Weifang D, Yongliang G (1999). Establishment of a highly metastatic human ovarian cancer cell line (HO-8910PM) and its characterization. J Exp Clin Cancer Res.

[6] Yabushita H, Ueno N, Sawaguchi K, Higuchi K, Noguchi M, Ishihara M (1989). Establishment and characterization of a new human cell-line (AMOC-2) derived from a serous adenocarcinoma of ovary. Nihon Sanka Fujinka Gakkai zasshi.

[7] Korch C, Spillman MA, Jackson TA, Jacobsen BM, Murphy SK, Lessey BA, Jordan VC, Bradford AP (2012). DNA profiling analysis of endometrial and ovarian cell lines reveals misidentification, redundancy and contamination. Gyn Onc.

[8] Mukhopadhyay A, Elattar A, Cerbinskaite A, Wilkinson SJ, Drew Y, Kyle S, Los G, Hostomsky Z, Edmondson RJ, Curtin NJ (2010). Development of a functional assay for homologous recombination status in primary cultures of epithelial ovarian tumor and correlation with sensitivity to poly (ADP-ribose) polymerase inhibitors. Clin Can Res.

[9] Drew Y, Mulligan EA, Vong WT, Thomas HD, Kahn S, Kyle S, Mukhopadhyay A, Los G, Hostomsky Z, Plummer ER, Edmondson RJ, Curtin NJ (2011). Therapeutic potential of poly(ADP-ribose) polymerase inhibitor AG014699 in human cancers with mutated or methylated BRCA1 or BRCA2. J Natl Cancer Inst.

[10] O Donnell RL, McCormick A, Mukhopadhyay A, Woodhouse LC, Moat M, Grundy A, Dixon M, Kaufman A, Soohoo S, Elattar A, Curtin NJ, Edmondson RJ (2014). The use of ovarian cancer cells from patients undergoing surgery to generate primary cultures capable of undergoing functional analysis. PLos One.

[11] Ahmed AA, Etemadmoghadam D, Temple J, Lynch AG, Riad M, Sharma R, Stewart C, Fereday S, Caldas C, Defazio A, Bowtell D, Brenton JD (2010). Driver mutations in TP53 are ubiquitous in high grade serous carcinoma of the ovary. J Path.

[12] Gorringe KL, Campbell IG (2009). Large-scale genomic analysis of ovarian carcinomas. Mol Oncol.

[13] Mackenzie R, Talhouk A, Eshragh S, Lau S, Cheung D, Chow C, Le N Cook LS, Wilkinson N, McDermott J, Singh N, Kommoss F, Pfisterer J (2015). Morphologic and Molecular Characteristics of Mixed Epithelial Ovarian Cancers. Am J Surg Path.

[14] Maloney KE, Norman RW, Lee CL, Millard OH, Welch JP (1991). Cytogenetic abnormalities associated with renal cell carcinoma. J Urol.

[15] Abramoff M, Magelhaes P, Ram S (2004). Image processing with ImageJ. Biophoton Int.

[16] Znojek P (2011).

[17] Diggle CP, Bentley J, Kiltie AE (2003). Development of a rapid, small-scale DNA repair assay for use on clinical samples. Nucleic Acids Res.

[18] Vichai V, Kirtikara K (2006). Sulforhodamine B colorimetric assay for cytotoxicity screening. Nat Protoc.

