# Supplementary material for: Functional characterisation of a novel ovarian cancer cell line, NUOC-1
Source: Oncotarget. 2017 Mar 1;8(16):26832–44. doi: 10.18632/oncotarget.15821 (PMC5432300; doi:10.18632/oncotarget.15821)
Supplement: Supplementary file 1 [file oncotarget-08-26832-s001.pdf]

Functional characterisation of a novel ovarian cancer cell line, NUOC-1

Supplementary Materials

Supplementary Table 1: STR profiling report in NUOC-1 cell line and 2 subpopulation derivatives – NUOC-1-A1 and NUOC-1-A2

Loci	NUOC-1		NUOC-1-A1		NUOC-1-A2	
TH01	7	8	7	8	7	8
TPOX	9	11	9	11	9	11
v WA	15		15		15	
CSF1PO	10	13	10	13	10	13
D16S539	9	13	9	13	9	13
D7S820	7	8	7	8	7	8
D13S317	8		8		8	
D5S818	11		11		11	
Amelogenin	X		X		X	

Supplementary Table 2: Copy number variations in NUOC-1 cell line and 2 subpopulation derivatives – NUOC-1-A1 and NUOC-1-A2. See Supplementary_Table_2
