# Supplementary material for: Functional characterisation of a novel ovarian cancer cell line, NUOC-1
Source: Oncotarget. 2017 Mar 1;8(16):26832–44. doi: 10.18632/oncotarget.15821 (PMC5432300; doi:10.18632/oncotarget.15821)
Supplement: Supplementary file 2 [file oncotarget-08-26832-s002.docx]

**Supplementary Table 2.** Copy number variations in NUOC-1 cell line and 2 subpopulation derivatives – NUOC-1-A1 and NUOC-1-A2.

| **Affected cell lines** | **Chromosome** | **Start position (bp)** | **End position (bp)** | **Size (kb)** | **Gain/Loss** | **Alteration in NUOC-1 parental cells** | **Additional comments** |
| --- | --- | --- | --- | --- | --- | --- | --- |
| NUOC-1-A1 and -A2 | 1 | 15808872 | 16299312 | 490.440 | Gain | YES | Focal |
| NUOC-1-A1 and -A2 | 1 | 17228131 | 18313386 | 1085.255 | Gain | YES | Focal |
| NUOC-1-A1 and -A2 | 1 | 21276854 | 22787711 | 1510.857 | Gain | YES | Focal |
| NUOC-1-A1 and -A2 | 1 | 94020247 | 95358322 | 1338.075 | Gain | YES | Focal |
| NUOC-1-A1 and -A2 | 1 | 109150720 | 110609288 | 1458.568 | Gain | YES | Focal |
| NUOC-1-A1 and -A2 | 1 | 146089268 | 147394004 | 1304.736 | Gain | YES | Focal |
| NUOC-1-A1 and -A2 | 1 | 151015495 | 152493154 | 1477.659 | Gain | YES | Focal |
| NUOC-1-A1 and -A2 | 1 | 153177373 | 155540660 | 2363.287 | Gain | YES | Focal |
| NUOC-1-A1 and -A2 | 1 | 199831111 | 201336984 | 1505.873 | Gain | YES | Focal |
| NUOC-1-A1 and -A2 | 1 | 204317650 | 204628601 | 310.951 | Gain | YES | Focal |
| NUOC-1-A1 and -A2 | 1 | 223587515 | 225232264 | 1644.749 | Gain | YES | Focal |
| NUOC-1-A1 | 1 | 244562587 | 245431939 | 869.352 | Gain | NO | Focal |
| NUOC-1-A1 and -A2 | 1 | 82154 | 113717127 | 113634.973 | CN LOH | YES | Large region of copy neutral LOH affecting the short arm right to the telomere in both NUOC-1-A1 and -A2 |
| NUOC-1-A1 and -A2 | 2 | 27589810 | 27812252 | 222.442 | Gain | YES | Focal |
| NUOC-1-A1 and -A2 | 2 | 28375772 | 29618678 | 1242.906 | Gain | YES | Focal |
| NUOC-1-A1 and -A2 | 2 | 37016156 | 39129522 | 2113.366 | Gain | YES | Focal |
| NUOC-1-A1 and -A2 | 2 | 42553315 | 44464629 | 1911.314 | Gain | YES | Complex amplification |
| NUOC-1-A1 and -A2 | 2 | 121868436 | 121925398 | 56.962 | Gain | YES | Focal |
| NUOC-1-A1 and -A2 | 2 | 122319019 | 122578634 | 259.615 | Gain | YES | Focal |
| NUOC-1-A1 and -A2 | 2 | 181900509 | 183752083 | 1851.574 | Gain | YES | Focal |
| NUOC-1-A1 and -A2 | 3 | 52012000 | 52021614 | 9.614 | Gain | NO | Focal |
| NUOC-1-A1 | 3 | 85057281 | 85200179 | 142.898 | Gain | NO | Focal |
| NUOC-1-A1 and -A2 | 3 | 98966922 | 100994578 | 2027.656 | Gain | YES | Focal |
| NUOC-1-A1 and -A2 | 3 | 149025861 | 149101811 | 75.950 | Gain | NO | Focal |
| NUOC-1-A1 and -A2 | 3 | 172087898 | 172477950 | 390.052 | Gain | YES | Focal |
| NUOC-1-A1 and -A2 | 4 | 16063055 | 16255566 | 192.511 | Gain | NO | Focal |
| NUOC-1-A1 and -A2 | 4 | 41263776 | 41807737 | 543.961 | Gain | YES | Focal |
| NUOC-1-A1 and -A2 | 4 | 74050725 | 75344134 | 1293.409 | Gain | YES | Focal |
| NUOC-1-A1 and -A2 | 4 | 139915883 | 140368070 | 452.187 | Gain | YES | Focal |
| NUOC-1-A1 and -A2 | 4 | 158221234 | 160607399 | 2386.165 | Gain | YES | Focal |
| NUOC-1-A1 and -A2 | 5 | 31188433 | 33302632 | 2114.199 | Gain | YES | Focal |
| NUOC-1-A1 and -A2 | 5 | 138498807 | 138951735 | 452.928 | Gain | NO | Focal |
| NUOC-1-A1 and -A2 | 5 | 158865362 | 160567322 | 1701.960 | Gain | YES | Focal |
| NUOC-1-A1 and -A2 | 5 | 170408962 | 171287243 | 878.281 | Gain | YES | Focal |
| NUOC-1-A1 and -A2 | 5 | 176409624 | 177377648 | 968.024 | Gain | YES | Focal |
| NUOC-1-A1 and -A2 | 6 | 10671764 | 11974582 | 1302.818 | Gain | YES | Focal |
| NUOC-1-A1 | 6 | 203878 | 33533404 | 33329.526 | Gain | YES | Large gain affecting most of the p arm to the telomere present in a prominent sub-clone. |
| NUOC-1-A1 and -A2 | 6 | 42478124 | 43631259 | 1153.135 | Gain | YES | Focal |
| NUOC-1-A1 and -A2 | 6 | 46546846 | 48205919 | 1659.073 | Gain | YES | Focal |
| NUOC-1-A1 and -A2 | 6 | 90941240 | 92493227 | 1551.987 | Gain | YES | Focal |
| NUOC-1-A1 and -A2 | 6 | 134574934 | 134973208 | 398.274 | Gain | YES | Focal |
| NUOC-1-A1 | 6 | 203878 | 134516019 | 134312.141 | Loss | NO | Large loss of material affecting the p arm to the telomere and most of the q arm but which is present in a very minor sub-clones in NUOC-1-A1 |
| NUOC-1-A1 and -A2 | 7 | 299843 | 1691600 | 1391.757 | Gain | YES | Focal |
| NUOC-1-A1 and -A2 | 7 | 84968982 | 85031803 | 62.821 | Loss | NO | Focal |
| NUOC-1-A1 and -A2 | 7 | 94865281 | 95697118 | 831.837 | Gain | YES | Focal |
| NUOC-1-A1 and -A2 | 7 | 104749474 | 105735118 | 985.644 | Gain | YES | Focal |
| NUOC-1-A1 and -A2 | 7 | 138157079 | 141421031 | 3263.952 | Gain | YES | Focal |
| NUOC-1-A1 | 8 | 29082965 | 146293414 | 117210.449 | Gain | NO | Large gain affecting some of the p arm and all of the q arm |
| NUOC-1-A1 | 8 | 39008680 | 40836593 | 1827.913 | Gain | NO | Complex amplification |
| NUOC-1-A2 | 8 | 39021272 | 43791691 | 4770.419 | Gain | YES | Complex amplification |
| NUOC-1-A1 and -A2 | 8 | 54628383 | 56171940 | 1543.557 | Gain | YES | Focal |
| NUOC-1-A1 and -A2 | 8 | 80812152 | 81640595 | 828.443 | Gain | YES | Focal |
| NUOC-1-A2 | 8 | 90576635 | 90958422 | 381.787 | Loss | YES | Focal |
| NUOC-1-A1 and -A2 | 8 | 119028915 | 120707600 | 1678.685 | Gain | YES | Focal |
| NUOC-1-A2 | 8 | 123928360 | 136097222 | 12168.862 | Gain | YES | Complex amplification affecting c-MYC |
| NUOC-1-A1 | 8 | 164984 | 29082965 | 28917.981 | CN LOH | NO | Large region of CN LOH affecting most of the p arm (p telomere - 29082965) in NUOC-1-A1. |
| NUOC-1-A2 | 9 | 46587 | 46386250 | 46339.663 | Loss | YES | Entire short arm loss of material in a minor sub-clone right to the p Telomere |
| NUOC-1-A1 and -A2 | 9 | 80747853 | 81083897 | 336.044 | Gain | YES | Focal |
| NUOC-1-A1 and -A2 | 9 | 122937656 | 124704304 | 1766.648 | Gain | YES | Focal |
| NUOC-1-A1 and -A2 | 9 | 139487092 | 139569261 | 82.169 | Gain | NO | Focal |
| NUOC-1-A1 and -A2 | 9 | 140034431 | 140262218 | 227.787 | Gain | NO | Focal |
| NUOC-1-A2 | 10 | 98087 | 39088829 | 38990.742 | Loss | YES | Entire short arm loss of material in the major clone right to the telomere. |
| NUOC-1-A1 and -A2 | 10 | 34342143 | 35980025 | 1637.882 | Gain | YES | Focal |
| NUOC-1-A1 and -A2 | 10 | 68078481 | 68114481 | 36.000 | Loss | NO | Focal |
| NUOC-1-A1 and -A2 | 10 | 72477417 | 74639217 | 2161.800 | Gain | YES | Focal |
| NUOC-1-A1 and -A2 | 10 | 108646505 | 108950282 | 303.777 | Gain | YES | Focal |
| NUOC-1-A1 and -A2 | 10 | 112270132 | 112522967 | 252.835 | Gain | YES | Focal |
| NUOC-1-A1 and -A2 | 11 | 556531 | 1398316 | 841.785 | Gain | YES | Focal |
| NUOC-1-A1 and -A2 | 11 | 34548906 | 34699269 | 150.363 | Gain | YES | Focal |
| NUOC-1-A1 and -A2 | 11 | 63431526 | 63787389 | 355.863 | Gain | NO | Focal |
| NUOC-1-A1 and -A2 | 11 | 64799729 | 65048535 | 248.806 | Gain | YES | Focal |
| NUOC-1-A1 and -A2 | 11 | 67856510 | 74636892 | 6780.382 | Loss | YES | Large region of loss of material |
| NUOC-1-A1 and -A2 | 11 | 117446945 | 118964661 | 1517.716 | Gain | YES | Focal |
| NUOC-1-A1 and -A2 | 11 | 47235918 | 51566909 | 4330.991 | CN LOH | YES | Copy neutral LOH on the p arm extending to the centromere in both NUOC-1-A1 and -A2 |
| NUOC-1-A1 and -A2 | 12 | 4013581 | 4206768 | 193.187 | Gain | YES | Focal |
| NUOC-1-A1 and -A2 | 12 | 105804590 | 107518636 | 1714.046 | Gain | YES | Focal |
| NUOC-1-A1 and -A2 | 13 | 49361407 | 112505203 | 63143.796 | Loss | YES | Loss of material affecting most of the q arm giving rise to complete LOH |
| NUOC-1-A1 and -A2 | 14 | 23556072 | 23585333 | 29.261 | Gain | YES | Focal |
| NUOC-1-A1 and -A2 | 14 | 49663643 | 50966820 | 1303.177 | Gain | YES | Focal |
| NUOC-1-A1 and -A2 | 14 | 71076334 | 71314581 | 238.247 | Gain | YES | Focal |
| NUOC-1-A1 and -A2 | 14 | 73271879 | 75434102 | 2162.223 | Gain | YES | Focal |
| NUOC-1-A1 and -A2 | 14 | 77019135 | 77810543 | 791.408 | Gain | YES | Focal |
| NUOC-1-A1 and -A2 | 14 | 103215234 | 103349176 | 133.942 | Gain | YES | Focal |
| NUOC-1-A1 and -A2 | 14 | 104545285 | 106103358 | 1558.073 | Gain | YES | Focal |
| NUOC-1-A1 and -A2 | 15 | 34929955 | 34979705 | 49.750 | Loss | YES | Focal |
| NUOC-1-A1 and -A2 | 15 | 70288607 | 72040774 | 1752.167 | Gain | YES | Focal |
| NUOC-1-A1 | 16 | 88165 | 35285582 | 35197.417 | CN LOH | NO | Emerging CN LOH across the entire p arm in NUOC-1-A1 right to the telomere. |
| NUOC-1-A1 and -A2 | 16 | 3805990 | 5724014 | 1918.024 | Gain | YES | Focal |
| NUOC-1-A1 and -A2 | 16 | 11837682 | 12353187 | 515.505 | Gain | YES | Focal |
| NUOC-1-A1 and -A2 | 16 | 65375762 | 65492295 | 116.533 | Gain | YES | Focal |
| NUOC-1-A1 | 16 | 66424235 | 90130152 | 23705.917 | Gain | NO | Gain of material affecting half of the q arm in NUOC-1-A1 right to the telomere in a minor sub-clone. |
| NUOC-1-A2 | 16 | 67625872 | 69210661 | 1584.789 | Gain | YES | Focal |
| NUOC-1-A2 | 16 | 85000226 | 85097945 | 97.719 | Gain | NO | Focal |
| NUOC-1-A1 and -A2 | 16 | 89475817 | 89667337 | 191.520 | Gain | YES | Focal |
| NUOC-1-A1 and -A2 | 17 | 455814 | 2027644 | 1571.830 | Gain | YES | Focal |
| NUOC-1-A1 and -A2 | 17 | 4278835 | 4501015 | 222.180 | Gain | YES | Focal |
| NUOC-1-A1 and -A2 | 17 | 35910735 | 36316479 | 405.744 | Gain | YES | Focal |
| NUOC-1-A1 and -A2 | 17 | 37771064 | 37886503 | 115.439 | Gain | YES | Focal |
| NUOC-1-A1 and -A2 | 17 | 38443493 | 38496320 | 52.827 | Gain | YES | Focal |
| NUOC-1-A1 and -A2 | 17 | 38833196 | 40744320 | 1911.124 | Gain | YES | Focal |
| NUOC-1-A1 and -A2 | 17 | 41424101 | 42699015 | 1274.914 | Gain | YES | Focal |
| NUOC-1-A1 and -A2 | 17 | 44165803 | 44350090 | 184.287 | Gain | YES | Focal |
| NUOC-1-A1 and -A2 | 17 | 45671506 | 46602432 | 930.926 | Gain | YES | Focal |
| NUOC-1-A1 and -A2 | 17 | 73904750 | 74134024 | 229.274 | Gain | YES | Focal |
| NUOC-1-A1 and -A2 | 17 | 75861653 | 76541994 | 680.341 | Gain | YES | Focal |
| NUOC-1-A2 | 17 | 78362923 | 80046280 | 1683.357 | Gain | YES | Focal |
| NUOC-1-A1 and -A2 | 17 | 45232068 | 45234651 | 2.583 | Loss | NO | Focal |
| NUOC-1-A1 | 17 | 72745164 | 81052230 | 8307.066 | Loss | NO | Loss of material giving rise to almost complete LOH . |
| NUOC-1-A1 | 17 | 46704536 | 72730234 | 26025.698 | CN LOH | NO | Emerging sub-clonal copy neutral LOH affecting part of the long arm |
| NUOC-1-A1 and -A2 | 18 | 23642772 | 24528949 | 886.177 | Gain | YES | Focal |
| NUOC-1-A1 and -A2 | 19 | 1163934 | 2565877 | 1401.943 | Gain | YES | Focal |
| NUOC-1-A1 and -A2 | 19 | 2805101 | 2907650 | 102.549 | Gain | YES | Focal |
| NUOC-1-A1 and -A2 | 19 | 11023434 | 11114352 | 90.918 | Gain | NO | Focal |
| NUOC-1-A1 and -A2 | 19 | 41773797 | 43513659 | 1739.862 | Gain | YES | Focal |
| NUOC-1-A1 and -A2 | 20 | 10497703 | 11830963 | 1333.260 | Gain | YES | Focal |
| NUOC-1-A2 | 20 | 29445644 | 33148758 | 3703.114 | Gain | YES | Large gain |
| NUOC-1-A1 and -A2 | 20 | 34010556 | 35068204 | 1057.648 | Gain | YES | Focal |
| NUOC-1-A1 and -A2 | 20 | 52114933 | 52516493 | 401.560 | Gain | YES | Focal |
| NUOC-1-A1 and -A2 | 20 | 60180124 | 62912463 | 2732.339 | Gain | YES | Focal |
| NUOC-1-A1 and -A2 | 21 | 43184093 | 43236119 | 52.026 | Gain | NO | Focal |
| NUOC-1-A1 and -A2 | 21 | 45733867 | 47754665 | 2020.798 | Gain | YES | Large gain |
| NUOC-1-A1 and -A2 | 22 | 20982876 | 22160301 | 1177.425 | Gain | YES | Focal |
| NUOC-1-A1 and -A2 | 22 | 29550379 | 30438447 | 888.068 | Gain | YES | Focal |
| NUOC-1-A1 | 22 | 35299912 | 37540187 | 2240.275 | Gain | NO | Large gain |
| NUOC-1-A1 and -A2 | 22 | 38447444 | 39159201 | 711.757 | Gain | YES | Focal |
| NUOC-1-A1 and -A2 | 22 | 41262852 | 41513774 | 250.922 | Gain | YES | Focal |
| NUOC-1-A1 and -A2 | 22 | 41911525 | 43782937 | 1871.412 | Gain | YES | Large Gain |
| NUOC-1-A1 | 22 | 29628615 | 51195728 | 21567.113 | CN LOH | NO | Emerging CN LOH affecting part of the q arm to the telomere in NUOC-1-A1 |
| NUOC-1-A2 | 22 | 16114244 | 43745197 | 27630.953 | CN LOH | NO | Emerging CN LOH affecting part of the q arm to the centromere in NUOC-1-A2 |
| NUOC-1-A1 and -A2 | X | 23672128 | 24049039 | 376.911 | Gain | YES | Focal |
